# Use of metabolic imaging to monitor heterogeneity of tumour response following therapeutic mTORC1/2 pathway inhibition

**DOI:** 10.1242/dmm.050804

**Published:** 2025-02-28

**Authors:** Stephanie Ling, Alex Dexter, Alan M. Race, Shreya Sharma, Gregory Hamm, Urszula M. Polanska, Rosetta Consortium Cancer Research UK, John F. Marshall, Zoltan Takats, Kevin Brindle, Mariia O. Yuneva, George Poulogiannis, Andrew D. Campbell, Owen J. Sansom, Richard J. A. Goodwin, Josephine Bunch, Simon T. Barry

**Affiliations:** ^1^ Imaging and Data Analytics, AstraZeneca, Cambridge CB2 0AA, UK; ^2^ National Physical Laboratory, Teddington TW11 0LA, UK; ^3^ Early Oncology, AstraZeneca, Cambridge CB2 0AA, UK; ^4^ https://www.cancergrandchallenges.org/rosetta; ^5^ Barts Cancer Institute, London EC1M 6AU, UK; ^6^ Imperial College London, London SW7 2AZ, UK; ^7^ The Rosalind Franklin Institute, Harwell Campus, Didcot OX11 0QS, UK; ^8^ CRUK Cambridge Institute, Cambridge CB2 0RE, UK; ^9^ Francis Crick Institute, London NW1 1AT, UK; ^10^ Institute of Cancer Research, London SW3 6JB, UK; ^11^ CRUK Scotland Institute, Glasgow G61 1BD, UK

**Keywords:** Mass spectrometry imaging, Imaging mass cytometry, Spatial multi-omics, PI3K, AKT, mTOR, Pharmacodynamic

## Abstract

The PI3K–mTOR–AKT pathway regulates tumour proliferation, gene expression and metabolism, but pathway inhibition induces heterogeneous feedback reactivation, limiting anti-tumour responses. Measuring heterogeneity of pathway inhibition in tissues using protein biomarker phosphorylation or location is challenging. An integrated multi-modal imaging workflow was developed to assess the heterogeneity of AZD2014 (mTORC1/2 inhibitor) response in a PTEN-null renal cancer model. Spatial responses of metabolite biomarkers were analysed by mass spectrometry imaging (MSI). Control and treated tumours were classified according to metabolite-defined regions enriched in control versus AZD2014-treated tumours, respectively. Noticeably, AZD2014-treated tumours retained regions similar to regions dominant in untreated tumours. Imaging mass cytometry analysis of protein biomarkers in ‘control-like’ regions following AZD2014 treatment showed reduced phospho-S6, indicating suppression, but retained high expression of the glucose transporter GLUT1. Increasing PI3K–AKT inhibition by combining with AZD8186 (PI3Kβ inhibitor) further decreased the control-like metabolic signature, showing PI3K-dependent resistance. This demonstrates that MSI-based workflows yield novel insights into the pharmacodynamic effects of mTORC1/2 inhibition in tumours, which classical biomarkers do not resolve. Coupling these workflows with spatial-omics approaches can deliver greater insights into heterogeneity of treatment response.

## Teaser

**Summary:** Monitoring spatial responses to drug treatment is challenging using conventional biomarkers. Using mass spectrometry imaging to guide other spatial biomarker platform analysis gives novel insights into heterogeneity of therapy response.

## INTRODUCTION

In all complex diseases, understanding phenotypic and functional heterogeneity at a molecular and cellular level is essential to guide treatment design, interpret response and resistance, and align preclinical model data to human disease segments.

The phosphoinositide 3-kinase (PI3K) pathway is among one of the most mutated pathways in cancer, with many molecules targeting different components of the pathway, including PI3Kα, PI3Kβ, PI3Kδ, AKT, mTORC1 and mTORC2 (Martini et al., 2014; Janku et al., 2018; Glaviano et al., 2023). Therapeutics targeting different nodes on the pathway have been developed and tested in clinical trials for solid tumour and haematological diseases (Janku et al., 2018). Regulation of PI3K–mTOR–AKT signalling is complex. The lipid kinases PI3Kα, PI3Kβ and PI3Kδ drive tumour cell growth in different contexts (Fruman et al., 2017). The *PIK3CA* gene encoding PI3Kα is commonly mutated (Samuels et al., 2004), rendering tumours more dependent on PI3Kα signalling (Liu et al., 2011; André et al., 2019), whereas loss of the tumour suppressor PTEN confers PI3Kβ dependency (Jia et al., 2008; Wee et al., 2008). In addition to inhibiting PI3Kα and PI3Kβ, the pathway can be blocked by inhibiting mTORC1 using rapamycin and its derivatives, which inhibit mTORC1 function in a kinase-independent manner (Boulay et al., 2004; Hurvitz et al., 2015); mTORC2 function using the mTORC1/2 kinase inhibitor AZD2014 (vistusertib) (Guichard et al., 2015); and AKT using kinase inhibitors ipatasertib and capivasertib (Davies et al., 2012; Lin et al., 2013). Each kinase controls different mechanisms: mTORC1 controls protein translation and amino acid signalling (Liu and Sabatini, 2020); mTORC2 controls activation of AKT (Manning and Toker, 2017); and AKT influences gene expression, cell survival, metabolism and proliferation (Fruman et al., 2017).

Investigating modulation of the PI3K–mTOR–AKT pathway in tumour tissue and consequences following treatment has been a major challenge. Insights into heterogeneity of response to therapeutics is critical as the pathway is subject to feedback reactivation and crosstalk through multiple mechanisms. PI3Kα inhibition leads to reactivation of signalling, or resistance through PI3Kβ activation (Costa et al., 2015; Juric et al., 2015), while PI3Kβ inhibition can result in activation of PI3Kα (Schwartz et al., 2015). Crosstalk or reactivation can be mediated by Erb family receptors, IGFR (also known as IGF1R), IR (also known as INSR), and activation of PI3K or ERK signalling (Chandarlapaty et al., 2011), as well as modulation of PTEN expression (Juric et al., 2015). Moreover, inhibiting AKT or mTORC2 can upregulate mTORC1 signalling, limiting potential efficacy (Dunn et al., 2022).

Mechanisms relating the PI3K–mTOR–AKT to pathway inhibitor response and resistance have been described *in vitro*. Therefore, validating these by mapping the heterogeneity of drug treatment effects in tumour tissue over time, from both preclinical *in vivo* or clinical samples, would improve understanding of potential therapeutic response. Currently, specific phospho (p)-biomarkers such as pAKT, pS6, pPRAS40, pNDRG1 and p4EBP1, nuclear translocation of the transcription factor FOXO, or fluorodeoxyglucose uptake by positron emission tomography imaging (Shi et al., 2022) inform on pathway activity. Although these biomarkers are useful, they cannot resolve more subtle spatial or heterogeneous treatment responses.

To address this challenge, a multi-omics workflow using mass spectrometry imaging (MSI) coupled with multiplex imaging mass cytometry (IMC) imaging was employed. This approach exploits MSI-generated insights using metabolite biomarkers and regional changes in metabolic function to visualise tissue heterogeneity and changes following drug treatment. The metabolite-based biomarkers are used in a ‘biology-independent mode’ to provide dense comparative spatial information with which to segment regions of tissues at different levels of resolution. The MSI signatures are overlaid with traditional antibody-based regional multi-biomarker analysis using IMC. This approach has been enabled as a result of a developments in MSI methodologies that can use spatial metabolite signatures to gain insights into metabolic function in tissues, and as a secondary output functional segmentation of tissues using metabolic signatures (Najumudeen et al., 2021; Kreuzaler et al., 2023; Vande Voorde et al., 2023). These advances are underpinned by methods that allow high-quality sample preparation and rapid analysis alongside data integration tools to merge different spatial omics datasets (Dannhorn et al., 2020; Murta et al., 2021; Race et al., 2021; Strittmatter et al., 2022a,b). This workflow was used to study the heterogeneity of response to the mTORC1/mTORC2 kinase inhibitor AZD2014 (Guichard et al., 2015) in a metabolic biomarker-agnostic way, revealing novel insights into the tumour response following treatment. This multi-omics workflow has the potential to provide new insights when applied to any therapeutic intervention in complex tumour tissues.

## RESULTS

### AZD2014 reduces PI3K–AKT–mTOR pathway biomarkers in PTEN-null 786-0 renal tumour xenografts

PI3K–mTOR–AKT pathway inhibition is commonly assessed by measuring changes in phosphorylation of pathway biomarkers such as S6, PRAS40, NDRG1 and AKT; however, insights derived from these biomarkers are limited because it is not clear what impact the biomarker modulation observed is having on other processes such as protein expression, gene transcription or metabolism (Guichard et al., 2015; Saxton and Sabatini, 2017). Moreover, spatial context is lost with analysis of biomarker modulation in tumour lysates. To assess heterogeneity of response to AZD2014 in preclinical tumour tissue, independent of traditional protein biomarkers, MSI was used. This technology platform detects endogenous metabolites and small-molecule therapeutics directly from the surface of tissue sections to produce spatially resolved signatures that can subsequently be used to segment tissues (Sugiura and Setou, 2010; Dexter et al., 2019; Sushentsev et al., 2023).

**Fig. 1.**
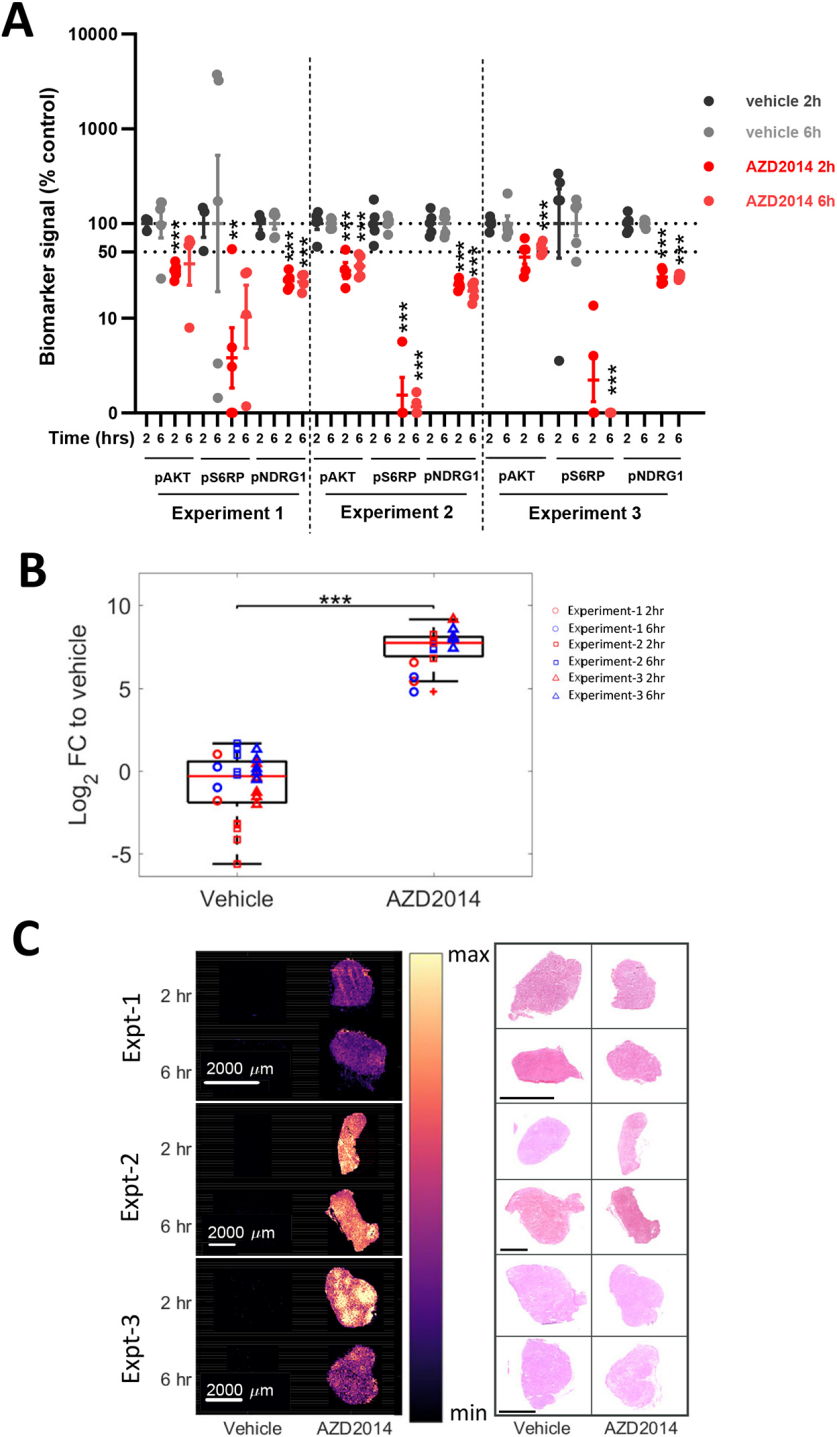
**Effect of AZD2014 on mTOR signalling and its biodistribution in tumours.** (A) Quantification of western blot analysis for biomarkers of PI3K and mTOR pathway activity in 786-0 tumours treated with AZD2014. Percentage reduction in phospho-signal for pAKT, pS6 and pNDRG1 relative to that in time-matched controls is shown for three independent experiments at the time points indicated, 2 and 6 h following AZD2014 treatment. **P<0.01, ***P<0.001 (two-way ANOVA). (B) Boxplot of the log2 fold change (FC) compared to vehicle in intensity of AZD2014 [M+H]^+^ (463.2452 m/z) from the desorption electrospray ionisation (DESI) mass spectrometry imaging (MSI) data. Boxplots show the log_2_ FC for the selected metabolite relative to the average of all vehicle intensities within the same experiment, with the red line representing the median, the box representing the 25% and 75% ranges, and the whiskers indicating the 1% and 99% range. Each individual tissue is then additionally represented by the coloured circles (Experiment-1), squares (Experiment-2) and triangles (Experiment-3). ***P<0.001 (two-sided *t*-test). (C) Single-ion images of AZD2014 [M+H]^+^ (463.2452 m/z) of selected treated and untreated tissues from the three different experiments, at the different timepoints (2 and 6 h post dosing) (left), alongside the corresponding Haematoxylin and Eosin (H&E) images for these tissues (right). Scale bars: 2000 µm. The tumour images shown in C are also used to exemplify representative analysis in Figs 2-5 and Figs S1-S5 to allow consistent visualisation of the analysis.

Treating tumour-bearing animals with the mTORC1/2 inhibitor AZD2014 (vistusertib) reduces growth of the PTEN-null renal tumour xenograft 786-0 (Zheng et al., 2015; Lynch et al., 2018). Lysates of tumours from each independent experiment showed consistent reduction of the PI3K–mTOR–AKT pathway biomarkers pAKT, pS6 and pNDRG1 at 2 and 6 h after treatment with AZD2014 (Fig. 1A). For acquisition of metabolic biomarker responses using MSI, representative tumours from three independent *in vivo* experiments were analysed (Figs S1 and S2). Omics platforms such as MSI can be subject to some run-to-run variability. Therefore, to account for this, samples from Experiment-1, -2 and -3 were analysed at different times in batches. MSI analysis and mapping was performed on 2 and 6 h AZD2014-treated and control tumours. AZD2014 was detected in all treated tumours (Fig. 1B), and the distribution of drug was relatively uniform across the tumours (Fig. 1C).

**Fig. 2.**
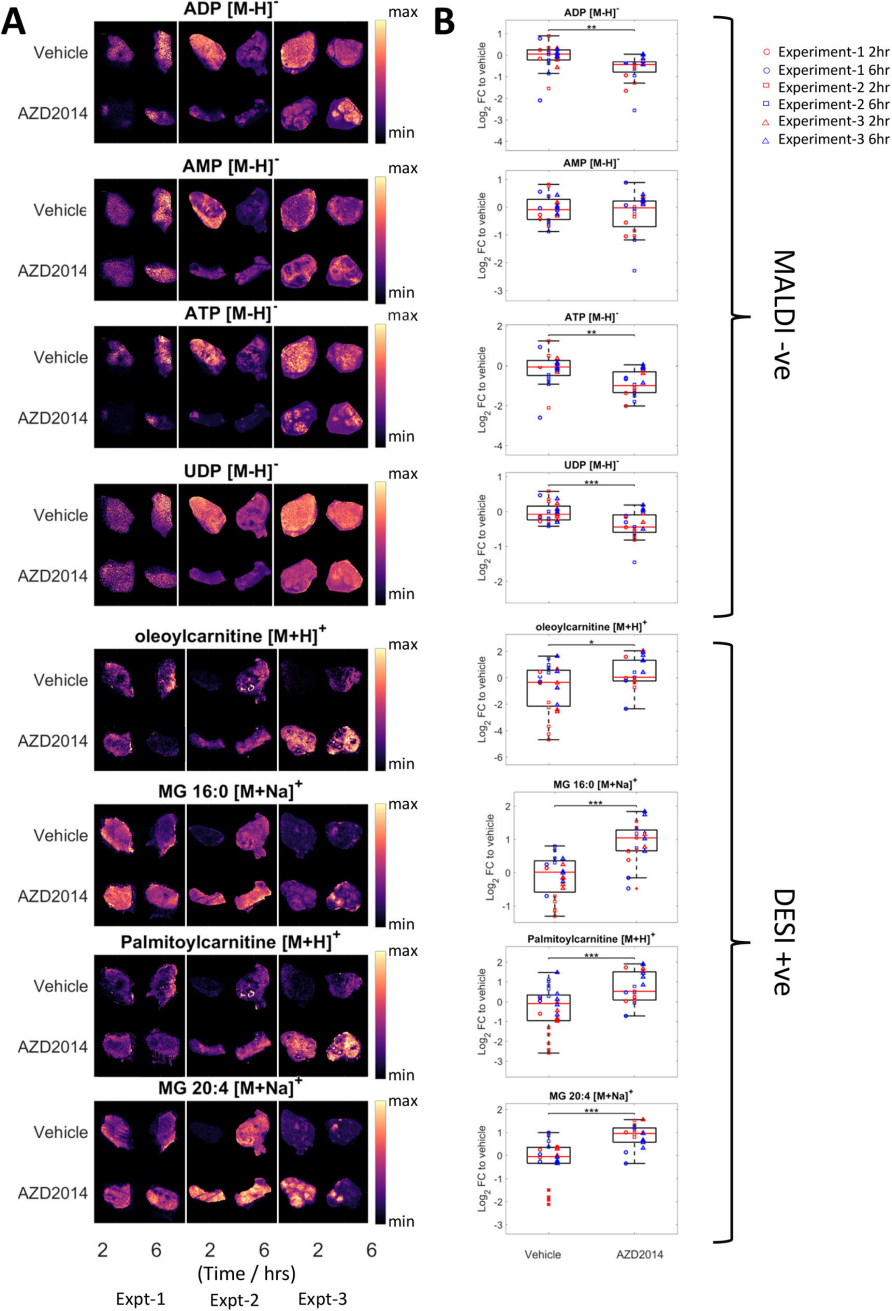
**Effect of AZD2014 treatment on tumour metabolism.** (A,B) Single-ion images (A) and boxplots (B) showing the log_2_ FC relative to vehicle of selected metabolites detected using matrix-assisted laser desorption–ionisation (MALDI) and DESI that are significantly different (*P*<0.05) between the treated and untreated tissues across the three different experiments. Boxplots show the log_2_ FC for the selected metabolite relative to the average of all vehicle intensities within the same experiment, with the red line representing the median, the box representing the 25% and 75% ranges, and the whiskers indicating the 1% and 99% range. Each individual tissue is then additionally represented by the coloured circles (Experiment-1), squares (Experiment-2) and triangles (Experiment-3). *P<0.05, **P<0.01 and ***P<0.001 (two-sided *t*-test). The tumour images shown in A are also used to exemplify representative analysis in Figs 1, 3, 4 and 5 and Figs S1-S5 to allow consistent visualisation of the analysis. ADP, adenosine diphosphate; AMP, adenosine monophosphate; ATP, adenosine triphosphate; MG, monoacylglycerol; UDP, uridine diphosphate.

**Fig. 3.**
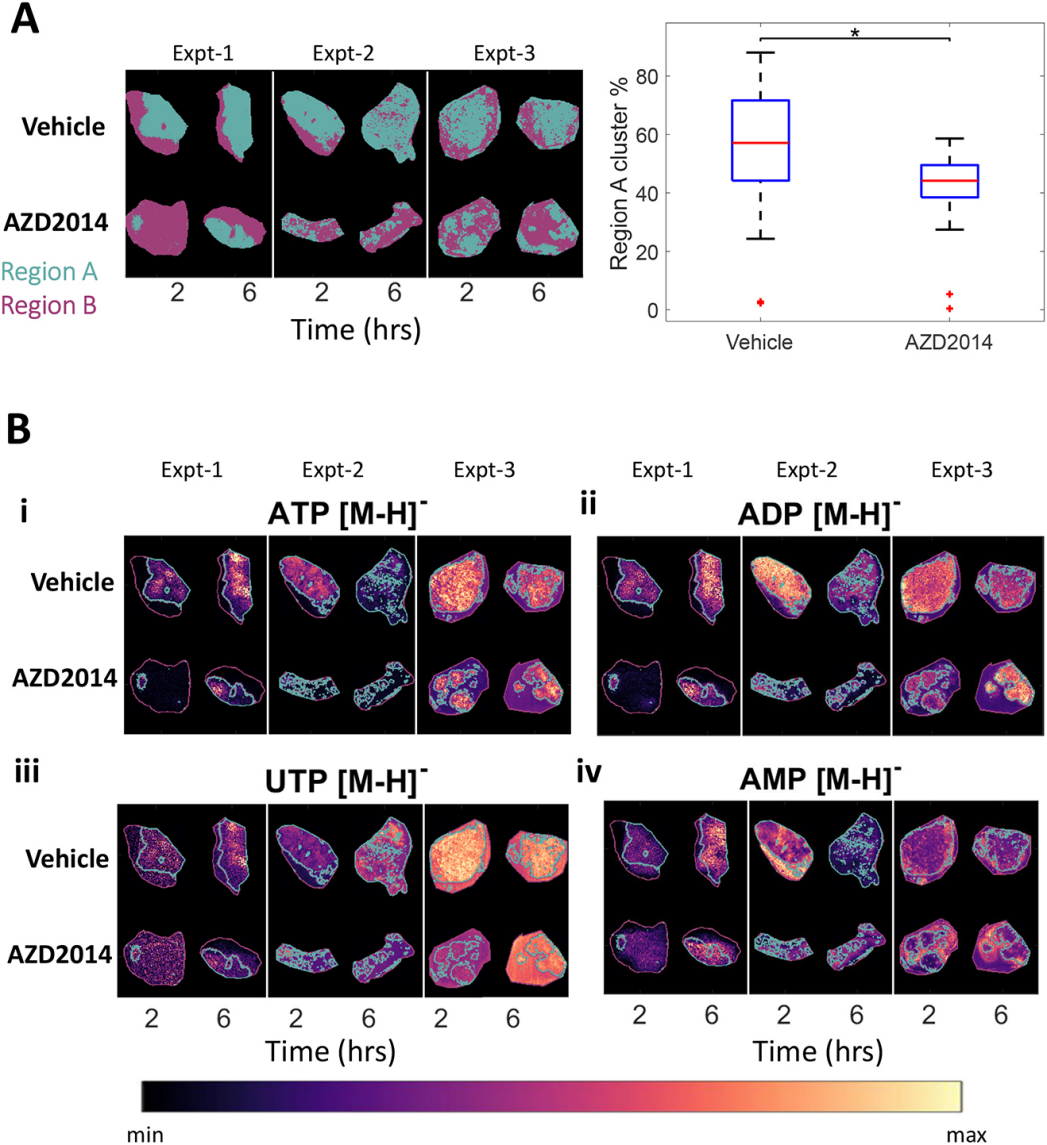
**Regional differences in the metabolic response to AZD2014 treatment.** (A) Unsupervised segmentation of metabolic differences in the treated and untreated tissues using neural network t-distributed stochastic neighbour embedding (t-SNE) and *k*-means clustering (*k*=2), and the corresponding boxplot of the percentage of the total tissue that is Region A (cyan), where the red line represents the median, the box represents the 25% and 75% ranges, and the whiskers indicate the 1% and 99% range. *P<0.05 (two-way ANOVA). (B) These clusters are then overlaid as an outline on the MALDI ion images from ATP (i), ADP (ii), uridine triphosphate (UTP; iii) and AMP (iv). The tumour images shown in B are also used to exemplify representative analysis in Figs 1, 2, 4 and 5 and Figs S1-S5 to allow consistent visualisation of the analysis.

**Fig. 4.**
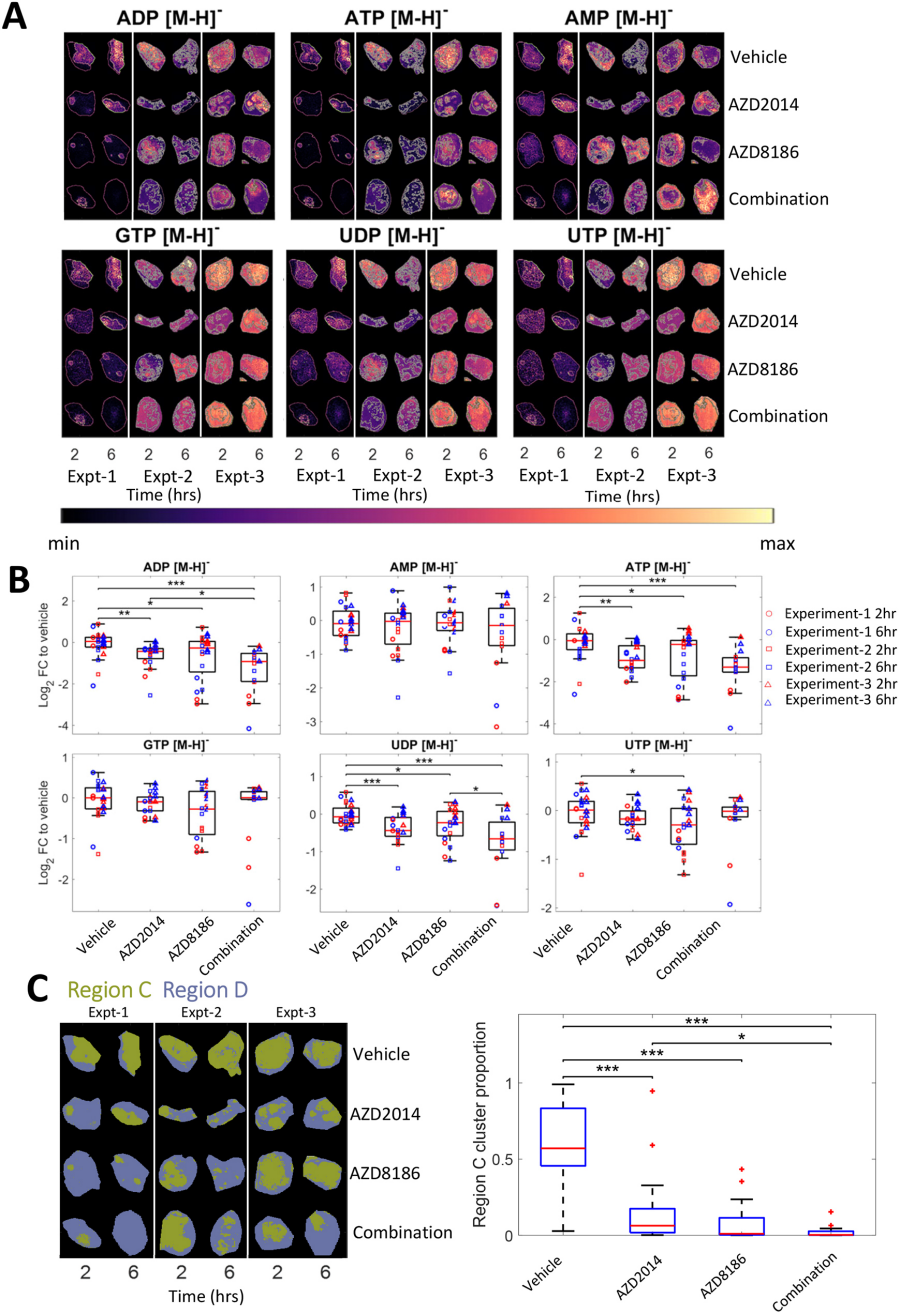
**Regional differences in metabolic response to AZD2014 and AZD8186 treatment.** (A-C) Selected single-ion images (A) outlined with the unsupervised segmentation (C) and corresponding boxplots (B), showing the log2 FC relative to vehicle of different metabolites detected using MALDI that are significantly different (*P*<0.05) between the singly treated, combination treated or untreated tissues across the three different experiments. These boxplots show the log2 FC for the selected metabolite relative to the average of all vehicle intensities within the same experiment, with the red line representing the median, the box representing the 25% and 75% ranges, and the whiskers indicating the 1% and 99% range. Each individual tissue is then additionally represented by the coloured circles (Experiment-1), squares (Experiment-2) and triangles (Experiment-3). *P<0.05, **P<0.01 and ***P<0.001 (two-sided *t*-test). (C) Unsupervised segmentation of metabolic differences in the treated and untreated tissues using neural network t-SNE and *k*-means clustering (left), and the corresponding boxplot (right) of the percentage of the total tissue that is Region C (green). *P<0.05 and ***P<0.001 (two-sided *t*-test). The tumour images shown in this figure are also used to exemplify representative analysis in Figs 1, 2, 3 and 5 and Figs S1-S5 to allow consistent visualisation of the analysis.

**Fig. 5.**
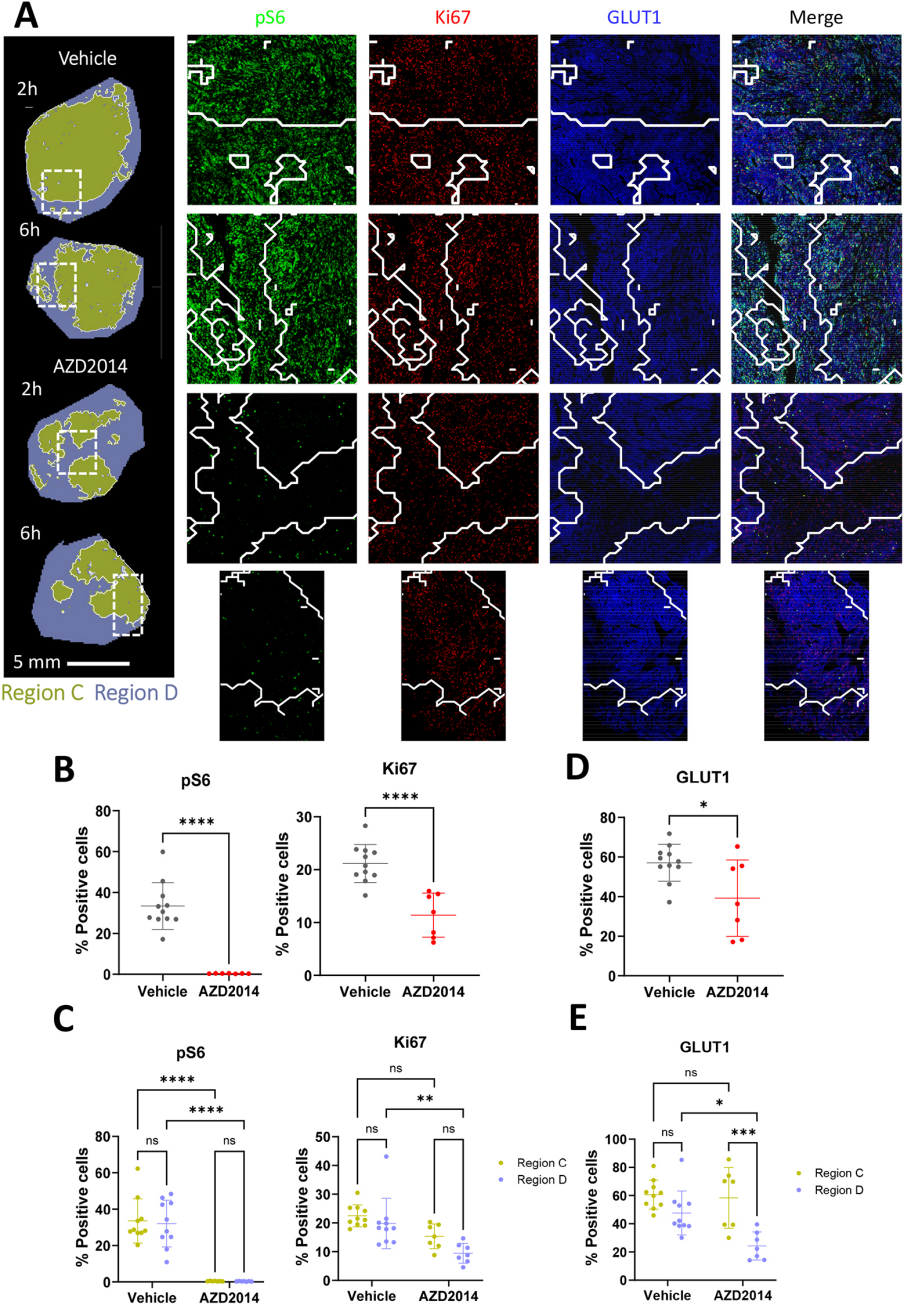
**Imaging mass cytometry (IMC) reveals regional response to AZD2014.** (A) Images from selected tissues of the unsupervised segmentation applied to the MSI data shown in Fig. 4C overlaid onto the IMC images. Example images of biomarkers of AZD2014 response, including pS6 (green), Ki67 (red) and GLUT1 (blue), across representative areas from Regions C and D. (B-E) Scatter plots of the intensities of these markers in the whole region analysed (B,D), and scatter plots with the data contained within the two different metabolic regions separated out as denoted by the corresponding colours (C,E). *P≤0.05, **P≤0.01, ***P≤0.001, ****P≤0.001 (two-way ANOVA). The tumours shown in this figure are also used to exemplify representative analysis in Figs 1-4 and Figs S1-S5 to allow consistent visualisation of the analysis.

### MSI analysis reveals AZD2014 treatment-associated changes in metabolite biomarkers

Metabolite changes following AZD2014 treatment were determined using both matrix-assisted laser desorption–ionisation (MALDI) and desorption electrospray ionisation (DESI) MSI as these two ionisation methods detect some of the same and some complementary metabolites. Differential detection of metabolites can be achieved depending on the mass spectrometry technique and polarity used. Using both MALDI and DESI in parallel enables greatest coverage of different types of metabolites. For example, nucleotides can be detected by MALDI, and lipids such as carnitine can be detected by DESI. The platform used and the polarity are indicated in each figure. The log_2_ fold change in metabolites in the AZD2014-treated groups relative to those in vehicle was compared (Fig. 2). The normalised combined data from each independent MSI analysis are shown in Fig. S3. To identify molecules modulated by AZD2014, two-sided *t*-tests were performed between the average fold changes per treated and control tissue (Fig. 2A,B). Across the tissue section, a large number of metabolites changed, which all contributed to the segmentation analysis. In particular, AZD2014 treatment significantly increased carnitine derivatives and glyceride derivatives, and reduced some nucleotides, including adenosine triphosphate (ATP) and adenosine diphosphate (ADP), but did not significantly change adenosine monophosphate (AMP). The distribution of these metabolites was heterogeneous, which is an insight that bulk liquid chromatography–mass spectrometry (LC/MS) would not deliver. Indeed, in treated tumours, some regions retained high levels of metabolites, such as ATP, suggesting less response to AZD2014 in those areas. These metabolites may therefore be pharmacodynamically responsive metabolites and could act as biomarkers of the effect of treatment.

### Segmentation using composite metabolite profiles differentiates tumour response

To explore whether metabolite signatures are informative regarding the heterogeneity of AZD2014, response data were analysed by dimensionality reduction using neural network t-distributed stochastic neighbour embedding (t-SNE) (Dexter et al., 2020 preprint) followed by *k*-means clustering analysis (*k*=2) applied to the data from each experiment individually (Fig. 3A). AZD2014-treated and untreated tumours showed two dominant classes of metabolite-defined clusters (Fig. 3) that were associated with specific metabolite signatures exemplified by molecules (all specific molecules described have been tentatively assigned by mass only) ATP, ADP and AMP (Figs S3 and S4). It is important to note that these were not the only metabolites to change within the total tissue analysis. Although there were some changes in absolute intensity of the metabolites detected between analytical runs, e.g. ATP, ADP, AMP and uridine triphosphate (UTP) (Fig. 3B) metabolites were detected with a similar relative abundance profile across the analytical runs, i.e. intra-run trends were similar across all runs, allowing valid run-to-run tissue segmentation. Fig. 3 shows representative plots of selected metabolites that changed on treatment and between regions, as well as examples of metabolites that did not change, to exemplify the method output and analytical workflow.

The cyan cluster (Region A) was dominant in control tumours, and the lilac cluster (Region B) was enriched in AZD2014-treated tumours (Fig. 3A). Therefore, Region B appears to define metabolically differentiated tumour regions modulated by AZD2014 treatment. ATP, and to a lesser extent ADP and AMP, were associated with these residual regions in AZD2014-treated tumours that resembled control tumours and might therefore represent metabolic resistance or lack of target modulation.

### Increasing PI3K pathway inhibition further reduces residual Region A in treated tumours

In PTEN-null tumours, signalling through the PI3Kβ isoform can drive PI3K–AKT activation (Wee et al., 2008; Hancox et al., 2015; Schwartz et al., 2015; Maynard et al., 2016; Lynch et al., 2018). Combining PI3Kα (BYL719) and PI3Kβ (AZD8186) inhibitors (Schwartz et al., 2015), or AZD2014 and the PI3Kβ inhibitor AZD8186 (Hancox et al., 2015; Lynch et al., 2018), can increase anti-tumour effects through greater pathway inhibition. To test whether the residual ‘resistant’ regions were PI3K–mTOR–AKT pathway dependent, the segmentation was re-run using a larger dataset that included additional tumours treated with AZD8186 and the combination of both AZD2014 and AZD8186. Again, data were segmented into two regions. In this larger combined segmentation analysis, the regions were annotated as Region C (green; higher in control tumours) and Region D (blue; higher in treated tumours) (Fig. 4). The segmentation identified similar regions to those identified as Regions A and B, in which changes in the same metabolites drive the segmentation and show similar changes in Region C versus D ratios following AZD2014 and AZD8186 treatment. The segmentation including both the AZD8186 and combination treatments showed an 84% overlap in pixel occupancy with the segmentation using AZD2014 and vehicle alone. Furthermore, the same metabolites were found to differentiate these regions, such as high levels of ATP in both the cyan cluster (Region A) and green cluster (Region C) (Fig. 3B and Fig. 4A), indicating that Region C is analogous to Region A, and Region B is analogous to Region D. Importantly, the greatest reduction in cluster size was seen in the combination-treated group, suggesting that the signatures relate to the degree of pathway inhibition, discriminating between responding and non-responding regions of the tumour.

### IMC biomarker analysis of differentially expressed proteins between blue and green clusters

To understand whether there are differences between Regions A/C and Regions B/D at the signalling or cellular level, IMC was used (Giesen et al., 2014). A panel of antibody-based biomarkers was measured to assess differences between the regions and pharmacodynamic changes within regions following AZD2014 treatment (Fig. 5A-E). For analysis, Region C versus Region D segmentation was used. Representative regions incorporating both green Region C and blue Region D from each vehicle- and AZD2014-treated tumour were analysed using a panel of antibodies against a range of biomarkers, including the pharmacodynamic biomarkers pS6 and pAKT (Table S1), as well as additional tumour cell and tumour microenvironment biomarkers. The integration of the MSI and IMC datasets into one overlaid image enabled alignment of metabolite-defined regions to changes in protein biomarkers. This shows the power of using integrated MSI and histochemistry-based analyses to guide greater insights into local regional changes in tissues.

Consistent with the western blot analysis of tumour lysates, in the regions of interest (ROIs), the levels of pS6 and Ki67 were reduced in the AZD2014-treated tumours (Fig. 5B). The biomarker responses were then segmented based on clustering of Regions C and D (Fig. 5C). In AZD2014-treated samples, pS6 was reduced equally in both regions, implying equivalent compound-mediated pathway suppression (Fig. 5C). However, despite Ki67 within the total ROI being reduced by AZD2014 treatment, it remained higher within Region A present in treated tumours. This further suggests that the Region A areas in treated tumours are a region of resistance in which AZD2014 did not achieve tumour cell growth arrest despite apparent modulation of pathway biomarkers.

**Fig. 6.**
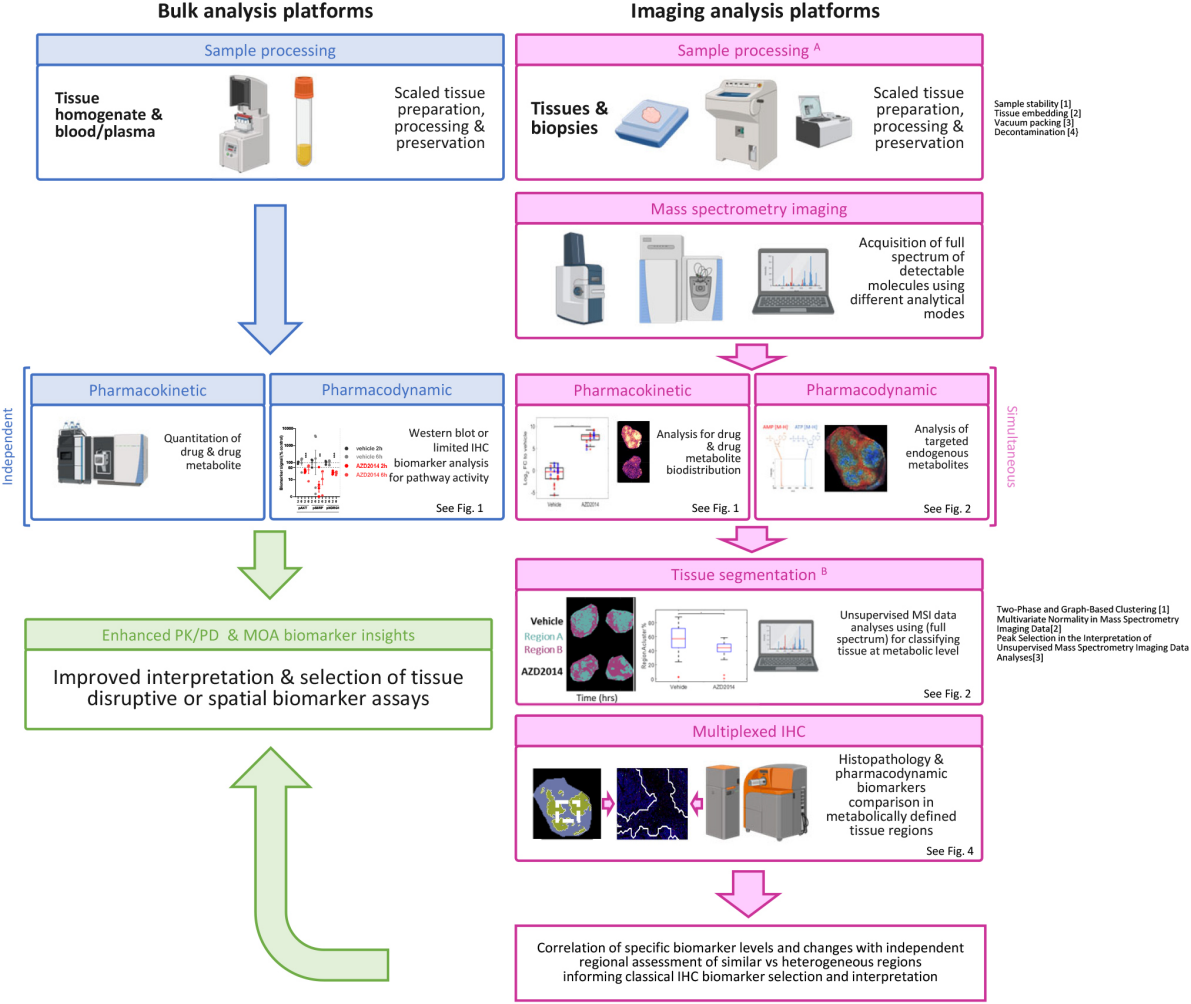
**Schematic of MSI-based analysis workflow for pharmacodynamic biomarker analysis.** Blue boxes: drug concentration is commonly measured in peripheral blood/plasma samples or from homogenised tissue. Biomarkers are commonly assessed by western blotting, enzyme-linked immunosorbent assay of homogenised tissue or immunohistochemistry (IHC) for protein biomarkers in independent samples. Pink boxes: image-based analysis underpinned by MSI is enabled by specific sample preparation and processing methodology (Swales et al., 2018; Dannhorn et al., 2020, 2021). Samples are scanned using MSI platforms in different analytical modes to collect a broad spectrum of metabolite biomarkers and detect drug distribution. This simultaneously generates a pharmacokinetic drug distribution and pharmacodynamic assessment of multiple metabolites, of which a subset of specific metabolites can inform on drug action. In addition, the full spectrum of metabolite biomarkers can be used to segment tissue and assess spatial responses to drug treatment using defined analytical workflows (Dexter et al., 2016, 2017; Murta et al., 2021). This segmentation is used as a cross reference to interpret spatial protein biomarker data generated by multiplex IHC. Collectively, these data give greater insight into the relevant biomarkers and heterogeneity of response, and contribute to improved interpretation of drug response. Green box: this multi-omics approach complements classical approaches and can be used to further filter the most informative biomarkers for specific preclinical or clinical biomarker assays. MAO, mode of action; PK/PD, pharmacokinetic/pharmacodynamic.

Within the broader biomarker panel, a number of other markers – such as CD31 (also known as PECAM1) and collagen 1 – were different between Regions C and D independent of treatment (Figs S5 and S6). However, notably, in the AZD2014-treated tumours, the expression of the glucose transporter GLUT1 (also known as SLC2A1) remained high in Region A even though there was generally a reduction following AZD2014 treatment in Region B (Fig. 5A,D,E). GLUT function, in particular the GLUT isoform GLUT4 (also known as SLC2A4), can be dynamically controlled by PI3K–mTOR–AKT signalling. Interestingly, here, GLUT1 expression was downregulated in regions responding to AZD2014, although GLUT1 is not commonly thought to be regulated by PI3K–AKT signalling. However, sustained GLUT1 expression was associated with lack of response to AZD2014, and could be a potential biomarker informing on levels of functional pathway inhibition and, in addition, indicate that regions of metabolic resistance involve GLUT1 function. Collectively, these data demonstrate that segmentation of tumour tissue using MSI-generated signatures gives different insights into tissue function following drug treatment, thus allowing discrimination of tissue phenotypes that classical biomarkers such as pS6 do not have sufficient power to resolve. As such, this represents a new workflow that can be applied to preclinical or clinical tissue to generate new insights into therapeutic response (Fig. 6).

## DISCUSSION

We have employed an MSI-guided segmentation workflow as a tool to investigate the heterogeneity of tumour response (Fig. 6). This workflow was used to assess the pharmacodynamic impact of the mTORC1/2 kinase inhibitor AZD2014 in the PTEN-null 786-0 renal carcinoma model. We found that specific metabolites – such as the nucleotides ATP, ADP and AMP – were reduced, and lipid metabolites – such as carnitine – were increased, following AZD2014 treatment. Analysis of changes in metabolite biomarkers to segment tumours and subsequent response to AZD2014 treatment identified residual regions of potential ‘resistance’ to AZD2014 treatment that mimicked regions dominant in untreated tumours. The residual metabolic signatures associated with the resistance regions appeared to be dependent on PI3K signalling, as they were further reduced with addition of the PI3Kβ inhibitor AZD8186. MSI signature-guided IMC analysis revealed that classical pharmacodynamic biomarkers of PI3K–mTOR signalling such as pS6 were reduced in the AZD2014-treated tumours, including in the residual ‘resistant’ regions. However, broader IMC analysis revealed that expression of the ubiquitous glucose transporter GLUT1 remained high in the residual regions that were metabolically similar to control tumours, and was not downregulated as seen in other regions of treated tumours. Hence, coupling MSI segmentation analysis with classical immunohistochemistry (IHC)-based biomarkers gave new insights into the response to AZD2014 that could not be identified using classical biomarker approaches.

To minimise intra- and inter-experiment artefact variability, samples were taken from three independent experiments that included AZD2014, AZD8186 and combination treatment. Analysis of these experiments showed that the methods applied are robust and can take into account run-to-run variability as well as study-to-study variability. This is important as independent runs are subject to variations in detection (intensity and coverage) of different metabolites. Despite this, segmentation analysis performed well across the whole sample set. Moreover, re-segmentation of the data that included additional samples with AZD2014-, AZD8186- and combination-treated tumours gave a similar result, suggesting that the approach can produce reproducible outcomes even when data are acquired in separate analytical runs over time or more variables are introduced. The data analyses also suggest that the metabolite profiles are consistent enough to build composite datasets, which would be important if deploying such approaches to assess clinical samples or looking across multiple experiments.

It should be noted that all metabolite identifications made using the approach described in this study are putative, based on the mass tolerances provided. These correlate with commonly reported metabolites identified using similar platforms to those used in this experiment. Fuller validation of metabolite identification would require incorporation of from-tissue fragmentation and comparison to analysis of labelled metabolite standards. Imaging of the identified metabolite would then require incorporation of homogeneously applied deuterated standard to the sample to confirm a consistent fragmentation. Data can also be correlated with bulk tissue homogenates or laser-captured tissue regions and LC/MS and nuclear magnetic resonance analysis. This is out of the scope of this study but can be performed for studies if absolute identification or validation of a metabolite is required.

Although the MSI-defined clusters gave rapid insights into the potential heterogeneity of response, they do not necessarily give insight into what drives the differences in response. As the MSI approach can be non-destructive (DESI-MSI), the segmentation guided by the MSI identified clear regions of interest that could then be analysed with specific protein imaging biomarkers. Here, we used IMC analysis, but other multiplex spatial imaging biomarkers would be suitable. Indeed, maximal insight is delivered by coupling the MSI segmentation with more detailed specific regional biomarker assessment. The coupled analysis indicated that biomarkers such as pS6 and pAKT, commonly used to assess pathway suppression by AZD2014, are not as discriminating as previously thought. These differences were unlikely to be related to drug distribution as AZD2014 was relatively homogenously distributed through the tumour, although amounts detected could vary between tumours. However, further pipeline development could allow full integration of MALDI and DESI datasets to investigate the detailed correlation between drug and all metabolites detected. Despite this, visual alignment of the drug distribution and tissue images did not indicate consistent specific differential distribution of drug between regions. The segmentation overlaid with the broad panel of IHC biomarkers enabled identification of other biomarkers, such as local Ki67 or sustained GLUT1 expression, which could have value in determining whether maximal pathway suppression has been achieved. These findings may challenge some assumptions that feedback reactivation of the pathway limits efficacy, showing that considering heterogeneous residual signalling is important. Within the IMC panel, pS6 was the most robust marker for measuring changes in pathway activation; changes in pAKT were less profound and did not reach significance, although there was a modest trend for reduction in treated tumours. This difference in dynamic range somewhat reflects the analysis performed on tumour lysates in which pS6 was also the most dynamic pathway biomarker. The reason for this is unclear. Phosphorylation of 4EBP1 (also known as eukaryotic translation initiation factor 4E-binding protein) was also modulated by treatment, although this lacked significance owing to variable phosphorylation of 4EBP1 in control tumours.

Using different omics approaches enables assumptions to be validated or challenged using multiple biomarkers. The PI3K–mTOR–AKT signalling network regulates many aspects of cellular functions (Martini et al., 2014; Manning and Toker, 2017; Glaviano et al., 2023), and it is impossible to explore all of these endpoints with specific antibody-based biomarkers in tissues. In addition to segmentation, the MSI data have potential to deliver novel insights that cannot be measured using standard approaches. Modification of specific metabolites – e.g. ATP, ADP and lipids – were associated with response to AZD2014 and AZD8186, which demonstrate the PI3K–AKT dependency of the effects. The association of GLUT1 expression with the regions showing less response to AZD2014 implies that part of the metabolic resistance profile results from sustained glucose uptake. The changes in GLUT1 expression seen here following AZD2014 treatment are intriguing. Although GLUT family members play an important role in mediating glucose uptake and metabolite exchange, it is GLUT4, rather than GLUT1, that is more commonly regulated by PI3K signalling (Ancey et al., 2018). Moreover, GLUT1 expression is seen where pS6 is reduced, suggesting that PI3K–AKT signalling is, to some extent, inhibited in that region. Therefore, although it is possible that GLUT1 downregulation is directly regulated by AZD2014 inhibition of PI3K–AKT signalling (Beg et al., 2017), other mechanisms might be involved, for example an association with reduction in proliferation or cell cycle progression or, conversely, a reduction in glucose requirement driving cell stress responses (Ancey et al., 2018; Shi et al., 2023). Likewise, the sustained expression could also be associated with PI3K-independent mechanisms such as cell stress, which result in sustained expression of GLUT1 in a subset of cells. Further work would be required to investigate this further.

Overall, this coupled approach can be invaluable for agents such as AZD2014, which inhibits multiple cellular mechanisms and is influenced by complex signalling networks that regulate the pathway (Costa et al., 2015; Schwartz et al., 2015; Hopkins et al., 2018, 2020; Mao et al., 2021). Access to high-throughput approaches that enable the diversity in therapeutic response to be assessed and then guide more focused and detailed biomarker assessment have been shown to be essential. A similar strategy has been successful in delineating metabolic lipid signatures that correlate with breast tumours dependent on PI3Kα signalling (Koundouros et al., 2020).

It is important to note that the experiments presented represent a large number of tissue samples analysed over a 4-year period. One of the primary reasons for performing these combined analyses was to ensure statistical and robust biological significance. Omics approaches can be subject to significant batch-to-batch variability, but comparison as a log_2_ fold change analysis relative to the vehicle treatment gave reproducibility in the outputs of the data analysis. This is vital if using MSI in larger and longitudinal preclinical or clinical studies. Any MSI-based spatial metabolomics tissue assessment has risk of inherent methodological and technical biases, but the methodology developments used in this workflow minimise these issues. First, sample preparation variation induced by tissue handling was minimised using optimised processing protocols (Swales et al., 2018; Dannhorn et al., 2020) by co-embedding all tissues in a randomised manner, followed by sectioning and vacuum sealing of slides and storing at ultra-low temperature (−80°C), enabling an unbiased MSI analysis and helping to preserve the stability of most endogenous metabolites (Fala et al., 2021). Combining DESI and MALDI, coupled with a high-resolution mass analyser, enables detection of a larger range of metabolite classes and allows orthogonal validation of mass annotations. Although IMC-based analysis of tissue can generate broad biomarker analyses, there is the major caveat of limited tissue area analysed (generally ∼2×2 mm) owing to throughput and time of analysis. Thus, combining MSI with IMC is an efficient method to select the most relevant ROI in the tumour and tissue microenvironment based on specific features. The approach described here could be expanded to create a ‘spatial metabolomics atlas’ of tumour molecular heterogeneity across cancer models with different genetic background. The data generated could be beneficial to better design pharmacodynamics studies involving different metabolic modulators, such as inhibitors of the AKT pathway.

In summary, we show a novel approach using MSI-based segmentation coupled with secondary biomarker analysis that can give novel insights into the functional heterogeneity of tumours and, moreover, how this can change in response to therapeutic intervention. Maximal insight is achieved when the approach is coupled with other complementary spatial biomarker approaches.

## MATERIALS AND METHODS

### 786-0 tumour growth

786-0 tumours were grown as previously described (Lynch et al., 2018). All animal experiments were performed according to the regulations of the Home Office UK. The 786-0 cells (5×10^6^ cells in RPMI serum-free medium mixed 50:50 with Matrigel™) were implanted into the flank of female SCID mice (AstraZeneca, Alderley Park, UK) between the ages of 8 and 12 weeks. Once tumours reached ∼200-500 mm^3^, animals were randomised into control and treatment groups. Tumour volume was calculated twice weekly from bilateral caliper measurements using the following formula:
(rmEqn1)

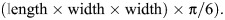


AZD8186 was generally formulated once weekly as a suspension in 0.5% hydroxypropyl methylcellulose (HPMC)/0.1% Tween™ 80 and dosed once or twice daily (0 and 6-8 h). AZD2014 was formulated as a suspension in 0.1% Tween™ 80. For combination dosing, AZD8186 and AZD2014 were co-formulated in 0.5% HPMC/0.1% Tween™ 80. Growth inhibition from the start of treatment was assessed by comparison of the geometric mean change in tumour volume for the control and treated groups. For pharmacodynamic protein biomarker analysis or MSI, tumours were snap frozen in liquid nitrogen.

### Pharmacodynamic studies

All pharmacodynamic biomarker analysis was performed as outlined in Lynch et al. (2018). Lysates were generated as follows: lysis buffer (Invitrogen, FNN0011), supplemented with phosphatase inhibitors 2 and 3 (Sigma-Aldrich, P5726 and P0044; 1:100), protease inhibitors (Roche, 11836145001; one tablet/50 ml) and DTT (1 mM) were added to each tumour in a Fastprep tube (MP Biomedicals, Santa Ana, CA, USA). The tumours were homogenised using a MP Biomedicals Fast Prep-24 machine. Samples were sonicated and centrifuged, and protein concentration was determined. All western blots were standardly loaded with equal amounts of protein. Protein was separated using SDS-PAGE, transferred to membranes and probed with the following primary antibodies: anti-pAKT S473 (Cell Signaling Technology, Danvers, MA, USA, 4060; 1:1000), anti-pNDRG1 T346 (Cell Signaling Technology, 5482; 1:1000) and anti-pS6RP S235/236 (Cell Signaling Technology, 4858; 1:1000). Equal transfer and loading was checked using Ponceau staining of membranes and by detecting vinculin levels (ab18058, Abcam, Cambridge, UK; 1:5000). Immune complexes were detected as described (Lynch et al., 2018). Binding was visualised using SuperSignal West Dura Chemiluminescent Substrate reagent (Pierce Thermo Scientific). Biomarker signals were quantified using Genetools software. Two-way ANOVA was used for statistical analyses. Vehicle controls were used for normalising biomarker signal for the treated samples, and change in phospho-signal relative to that of controls was represented. As part of the standard assay procedure, signal in individual samples was not normalised to total target protein. Raw western blot images are shown in Fig. S7.

### MSI analysis

As published previously (Dannhorn et al., 2020; Swales et al., 2018), tumour pieces were snap frozen and embedded randomised into blocks in a hydrogel of 7.5% HPMC/2.5% polyvinylpyrrolidone (two samples from each group in Experiment-1 were embedded in gelatine) before cryo-sectioning at 10 μm thickness with a CM1950 cryostat (Leica, Nussloch, Germany). Sections were thaw mounted onto Superfrost or conductive indium tin oxide-coated glass slides and dried using a flow of compressed air, then vacuum packed in a slide mailer and stored at −80°C (2). For MSI analysis, slides were thawed to room temperature before being unpacked to avoid analyte delocalisation through moisture condensation on the chilled slide surface.

MALDI-MSI analysis was carried out using a RapifleX MALDI-ToF Tissuetyper instrument (Bruker Daltonik, Bremen, Germany). Slides were first matrix coated with 10 mg/ml 9-aminoacridine in 80% MeOH applied in six passes using a TM-sprayer (HTX-Technologies, Chapel Hill, NC, USA) with the following parameters: temperature, 75°C; flow rate, 0.08 ml/min, with nitrogen pressure set to 6 psi; velocity, 1200 mm/min; 3 mm track spacing; nozzle height, 40 mm using criss-cross pattern. Image acquisition was done in negative mode at a spatial resolution of 50 µm, with 400 shots per pixel and a mass range of 60-1000 m/z.

DESI-MSI was performed on an automated 2D DESI stage (Prosolia, Indianapolis, IN, USA) equipped with a custom-built sprayer assembly (Swales et al., 2018) mounted to a Q-Exactive Plus instrument (Thermo Fisher Scientific, Bremen, Germany). Analysis was performed in positive-ion mode on one section and negative-ion mode on the sequential section in full-scan mode between 70 and 1000 m/z with 60 µm spatial resolution, SLens setting of 75, capillary temperature of 320°C, and a mass resolution of 70,000 at 200 m/z in profile ion mode. Automatic gain control was turned off. Electrospray solvent was methanol/water (95:5 v/v) at a flow rate of 1.5 μl/min, a spray voltage of ±4.5 kV and nebulising gas pressure of 6.5 bar (Nitrogen N4.8, BOC Gases, Woking, UK). Solvent was supplied using an Ultimate 3000 standalone nanoLC pump (Thermo Scientific Dionex, Sunnyvale, CA, USA).

The DESI-MSI .raw files were converted into .mzML files using ProteoWizard msConvert (Adusumilli et al., 2017) (v.3.0.4043), subsequently compiled to .imzML files [imzML converter (Race et al., 2012) v.1.3] and uploaded to SCiLS™ Lab MVS Premium 3D version 2021a (Bruker Daltonics, Bremen, Germany) for analysis. Root mean square-normalised mean peak intensity was calculated for each tissue. Further data analysis was performed using SpectralAnalysis (Race et al., 2016) and custom MATLAB scripts (Version 2019b; MathWorks, Natick, MA, USA). Data were first pre-processed using interpolation rebinning (bin width, 0.001 Da), a mean spectrum was created for each experiment, and peaks were detected using a gradient approach. These peaks were then tentatively assigned by accurate mass against the Human Metabolome Database (Wishart et al., 2007) using custom MATLAB scripts (Murta et al., 2021) using a 300 ppm inclusion window, a mass accuracy below 70 ppm, and adducts of [M−H]^−^ and [M+Cl]^−^ for MALDI negative, and 10 ppm inclusion window, a mass accuracy below 10 ppm, and adducts of [M+H]^+^, [M+Na]^+^ and [M+K]^+^ for DESI positive. Identification and annotation is tentative by mass only, with large tolerance, and additional experiments have been conducted in other studies and by other groups to increase confidence in this annotation (Tucker et al., 2019; Miyamoto et al., 2016; Strittmatter et al., 2022b). Following this initial processing, a novel workflow was developed to analyse the combination of data from the three experiments performed over a long period of time. First, for this integrated analysis, only peaks common to all three *in vivo* experiments were selected for subsequent analysis. Each image was generated by summing the intensities under the peaks detected, then thresholding to the 95th percentile (calculated across all tissues in a given experiment). The mean intensity for each ion per tissue was then calculated and normalised to the average intensity across all of the vehicle treatments in the corresponding experiment. The log2 fold change of this normalised intensity was then calculated, molecules were matched between the different experiments, and boxplots of these matched molecules were generated for all tissues in all experiments. Statistical significance of the differences between the different treatments was tested using a two-sided *t*-test, and pairs of significant differences were then plotted onto the corresponding boxplots. Clustering was performed on all data within a single experiment using *k*-means clustering (*k*=2) applied to the results of dimensionality reduction using neural network t-SNE (Dexter et al., 2020 preprint) on the peak picked data. The clusters were then manually matched together based on their corresponding upregulation and downregulation of the same metabolites. Raw data for metabolites detected are provided in Table S2.

### IMC preparation and analysis

The slides used for DESI negative mode imaging from Experiment-3 were then fixed and stained with the panel of antibodies shown in Table S1 for IMC with the Hyperion system [Standard Biotools (formerly known as Fluidigm), San Francisco, CA, USA] according to the manufacturer's guidelines. To stain slides with labelled antibodies, slides were fixed in 4% paraformaldehyde, washed and permeabilised for 5 min using 1× casein solution containing 0.1% Triton X-100, washed. Then, blocking solution was applied for 30 min, the slides were washed, and an antibody panel (Table S1) was applied and incubated at 4°C overnight. Slides were washed again, then DNA Ir-intercalator (Standard Biotools; diluted 1:400 in PBS) was applied for 30 min, before the slides were dipped in deionised water and dried. All washing steps consisted of 3×5 min in fresh PBS. Unless stated otherwise, all steps were performed at room temperature.

Images with ROIs of 2×2 mm covering representative regions of A and B in each tissue were acquired with a laser power of 6 db, frequency of 200 Hz and pixel size of 1 µm. Antibodies from other suppliers were custom conjugated with labelling kits from Standard Biotools according to the manufacturer's protocols. All antibodies were validated in house using conventional IHC as well as corresponding IMC staining and assessed by a veterinary pathologist. IMC data were analysed using the Halo HighPlex v3.02 module (Indica Laboratories, Albuquerque, NM, USA). Cell segmentation and thresholds were optimised manually. Nuclear segmentation was performed using the 191Ir DNA intercalator channel with the following parameters: nuclear contrast threshold, 0.49; minimum nuclear intensity, 0.09; nuclear segmentation aggressiveness, 0.65; nuclear size, 20-571 μm^2^; nuclear holes were not filled. Cytoplasm detection was performed assuming a concentric expansion from the cell nucleus with maximum cytoplasm radius of 4 μm. Maximum cell size was set to 600 μm^2^.

MSI and IMC data were registered via an affine transform using a modified version of Biquinho. MSI cluster maps were then transformed to the IMC space using the fitted affine transform (the inverse to the IMC to MSI process described in Strittmatter et al., 2022a), enabling each IMC pixel to be labelled with the MSI cluster (e.g. Region A, B, C or D). This then enabled the quantification of the intensity, as well as the number of (positive) cells, for each IMC marker within each MSI cluster.

## Supplementary Material

10.1242/dmm.050804_sup1 Supplementary information

Table S2. Raw data for metabolites detected
