# Supplementary material for: Use of metabolic imaging to monitor heterogeneity of tumour response following therapeutic mTORC1/2 pathway inhibition
Source: Dis Model Mech. 2025 Feb 28;18(2):DMM050804. doi: 10.1242/dmm.050804 (PMC11892681; doi:10.1242/dmm.050804)
Supplement: Supplementary information [file dmm-18-050804-s1.pdf]

Experiments

Experiment	Time after final dose	Experiment-1				Experiment- 2				Experiment-3			
Group		Vehicle	AZD8186	AZD2014	Combination	Vehicle	AZD8186	AZD2014	Combination	Vehicle	AZD8186	AZD2014	Combination
Number of samples	2h	2	5	5	4	5	4	4	1	5	4	2	1
	6h	2	5	4	2	5	5	3	3	5	4	5	1
Date of MALDI-MSI acquisition		2017/2018				2019/2020/2021				2019			

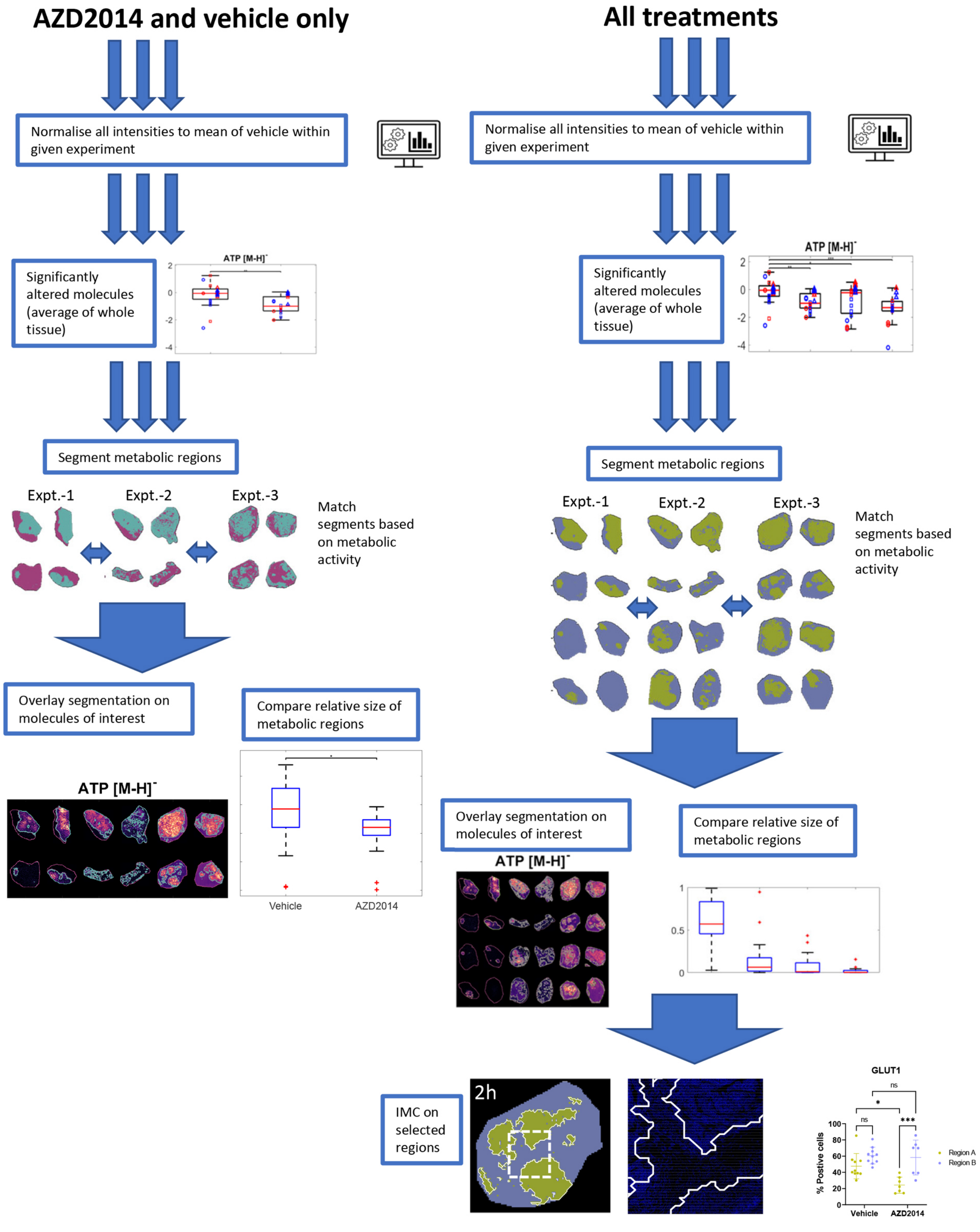

Disease Models & Mechanisms • Supplementary information

Fig. S1. Schematic of the overall workflow of data acquisition and analysis. Study flowchart presenting the processing of the MSI/IMC data within an individual experiment, metabolic segmentation, matching of features between the experiments, and subsequent data analysis of the differences between treatments. The initial analysis on just vehicle and AZD2014 tissues in Figures 1-3 is shown on the left, and the additional analysis including AZD8186 and combination therapy is shown on the right. For ease of visualisation of data representations the same tumours images are shown in multiple Figures (Figs. 1-5 in the main manuscript and Supp. Figs. 1-5).

Disease Models & Mechanisms • Supplementary information

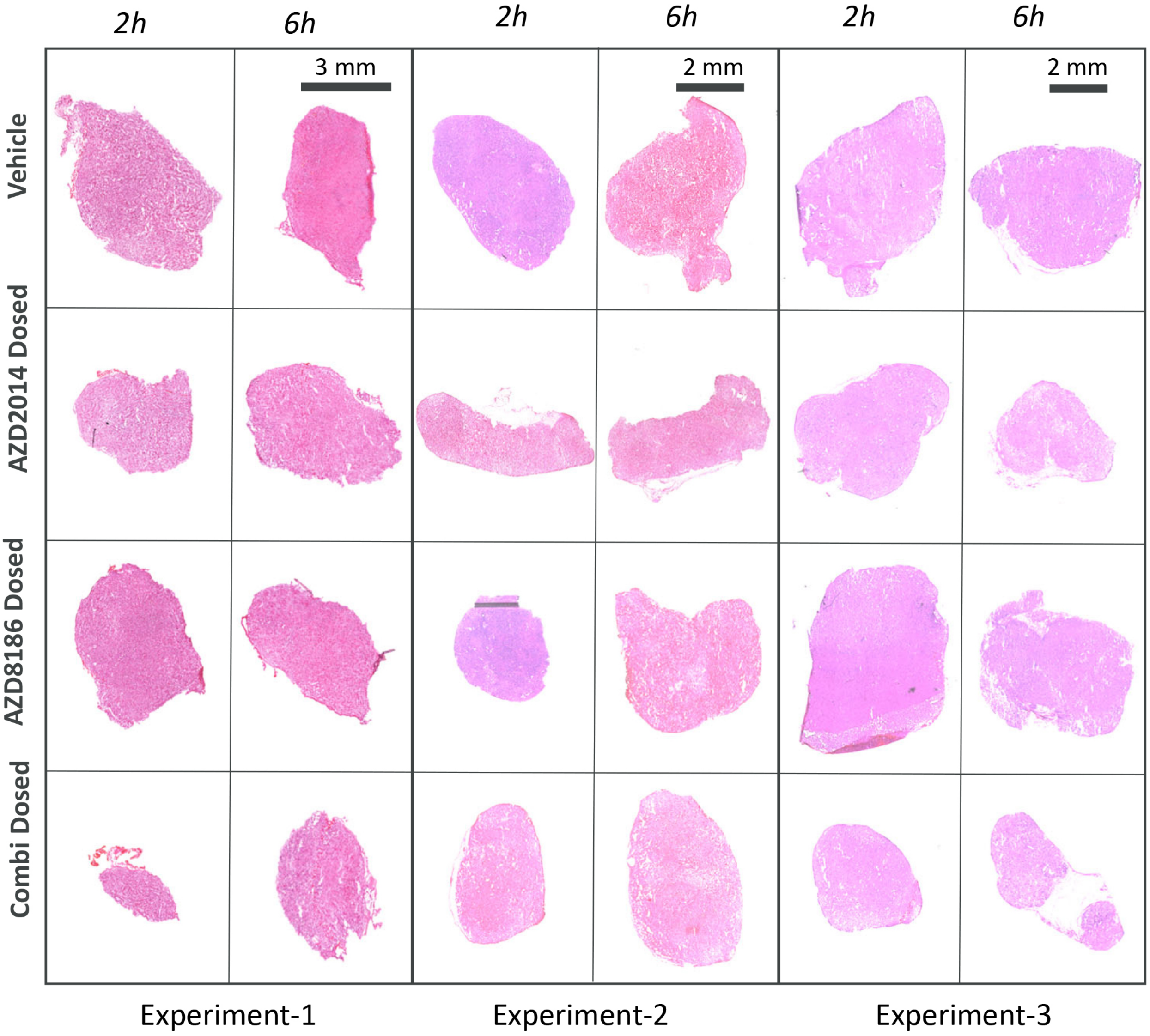

Fig. S2. Representative H&E images. H&E-stained sections of vehicle, AZD2014, AZD8186 and combination treated 786-O tumours from each of the different experiments and timepoints. Consecutive tissue sections that underwent MSI analysis. For ease of visualisation of data representations the same tumours images are shown in multiple Figures (Figs. 1-5 in the main manuscript and Supp. Figs. 1-5).

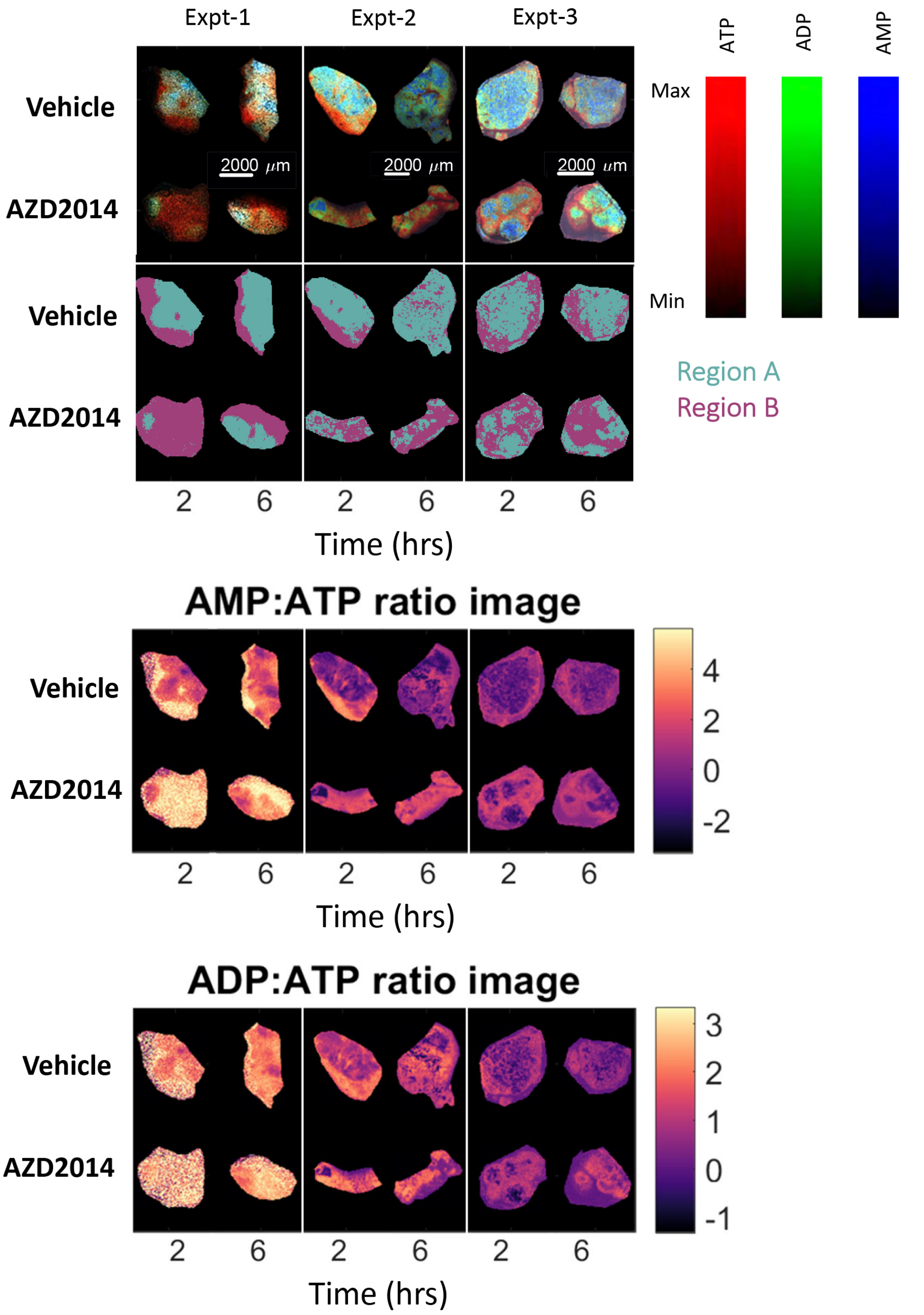

Fig. S3. Adenosine ribonucleotides across regions of metabolic heterogeneity. (A) Overlay of ion images of ATP (red, *m/z*505.989, $[M-H]^-$), ADP (green, *m/z* 426.022, $[M-H]^-$), and AMP (blue, *m/z* 346.056, $[M-H]^-$) from representative vehicle and treated tissues across the three experiments and two timepoints. Below are shown results from the corresponding unsupervised segmentation using neural network t-SNE and k-means clustering (k = 2) for the corresponding tissues describing regions A and B. Below this are the $\log_2$ ratio images of AMP:ATP and ADP:ATP respectively. For ease of visualisation of data representations of the same tumours images are shown in multiple Figures (Figs. 1-5 in the main manuscript and Supp. Figs. 1-5).

Disease Models & Mechanisms • Supplementary information

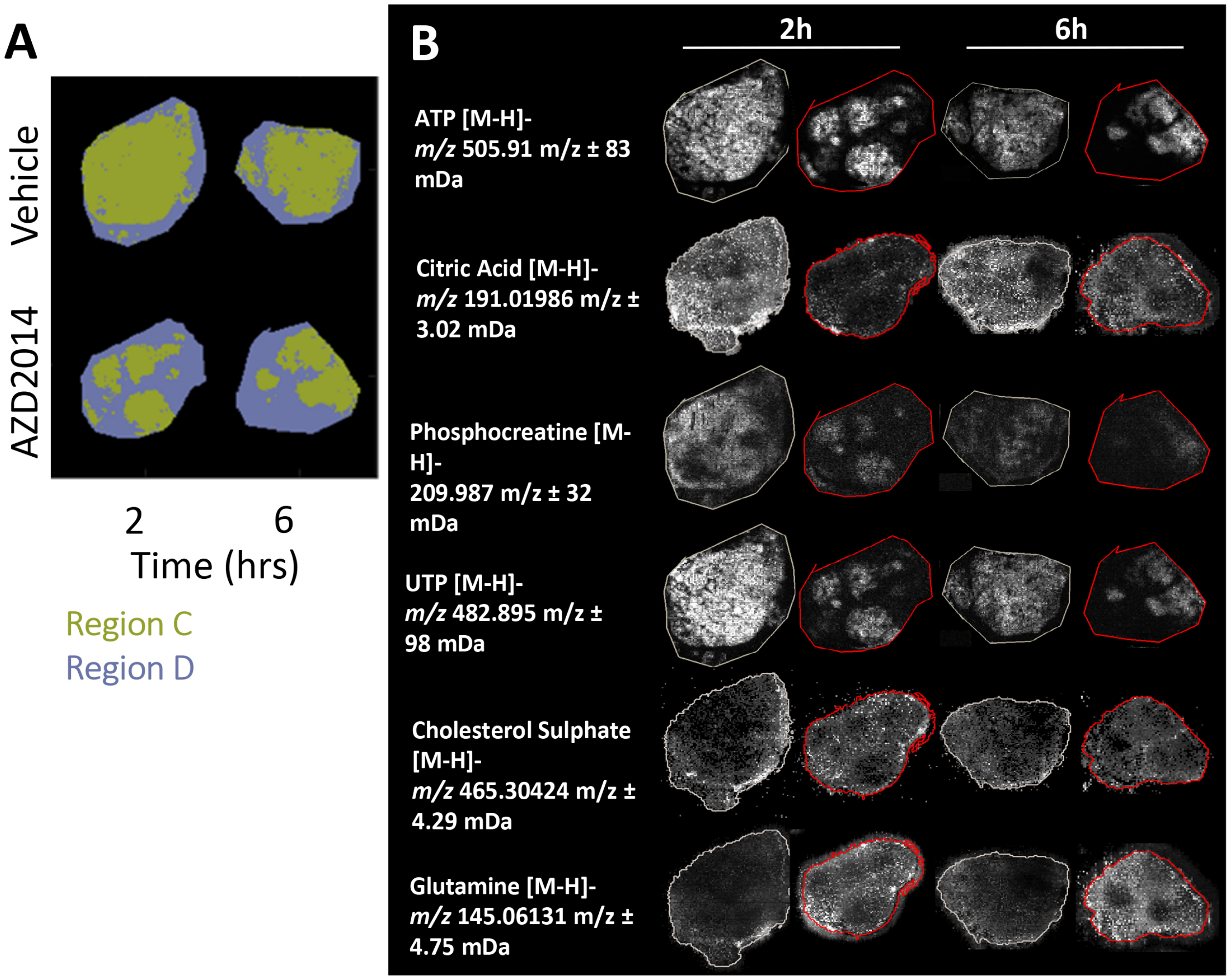

Fig. S4. Examination of metabolic features in regions A and B. (A) Clustering results showing Regions C and D from tissues in study 3. (B) Example images of metabolites differing between region C and D in study 3 acquired using different mass spectrometry imaging instruments. ATP,phosphocreatine, and UTP are detected by MALDI in negative polarity. Citric acid, cholesterol sulphate and glutamine are detected by DESI in negative polarity. For ease of visualisation of data representations of the same tumours images are shown in multiple Figures (Figs. 1-5 in the main manuscript and Supp. Figs. 1-5).

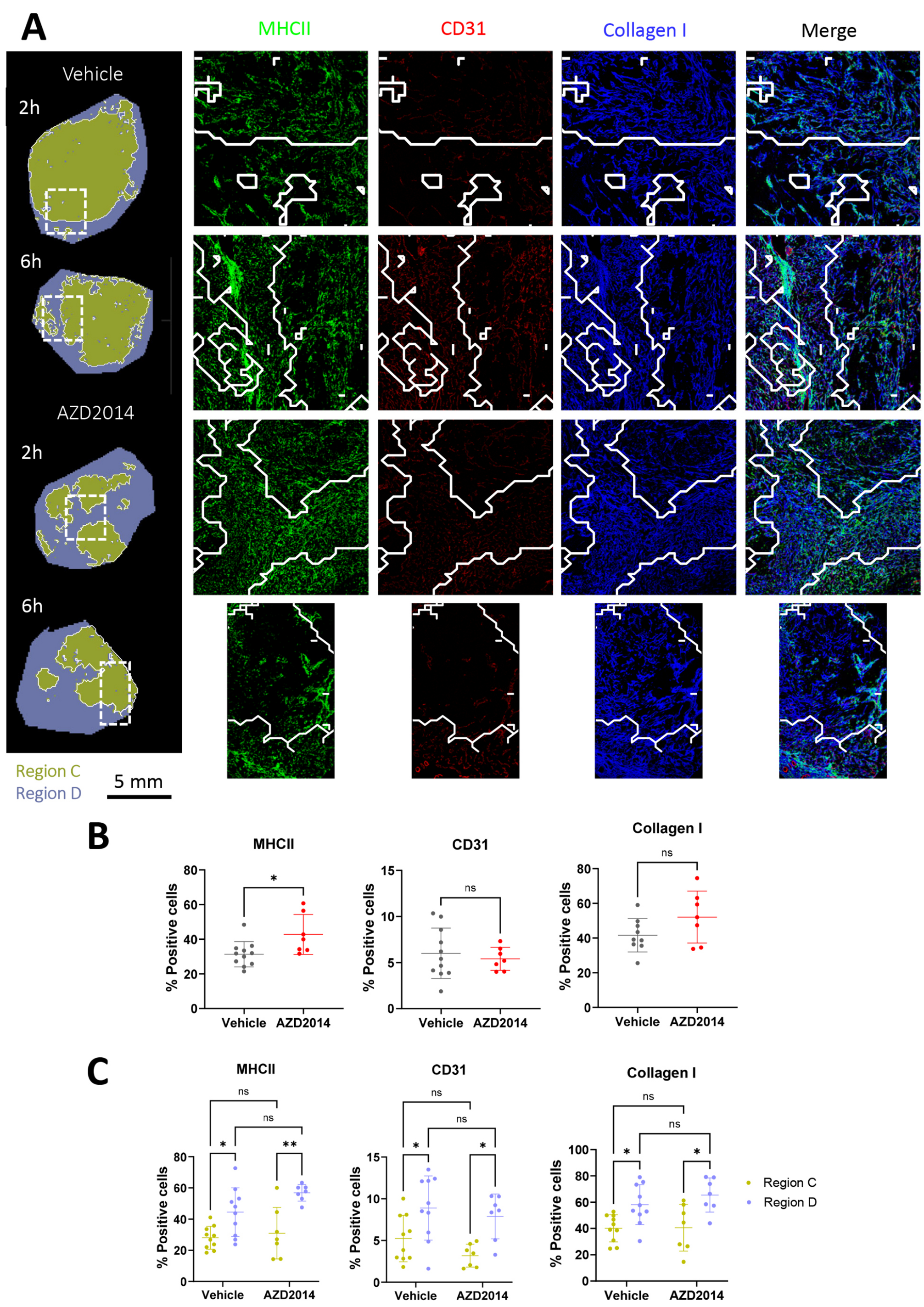

Fig. S5. Tissue phenotype differences between regions A and B. (A) Example images of markers that differ between regions C and D, including CD31, MHCII and collagen I in 2h treated AZD2014 treatment and 6h AZD2014 treatment. (B) Effect of AZD2014 treatment on MHCII, CD31 and Collagen expression. (C) Difference in MHCII, CD31 and Collagen I between regions C and D. Size bar 5mm. For ease of visualisation of data representations of the same tumours images are shown in multiple Figures (Figs. 1-5 in the main manuscript and Supp. Figs. 1-5).

Disease Models & Mechanisms • Supplementary information

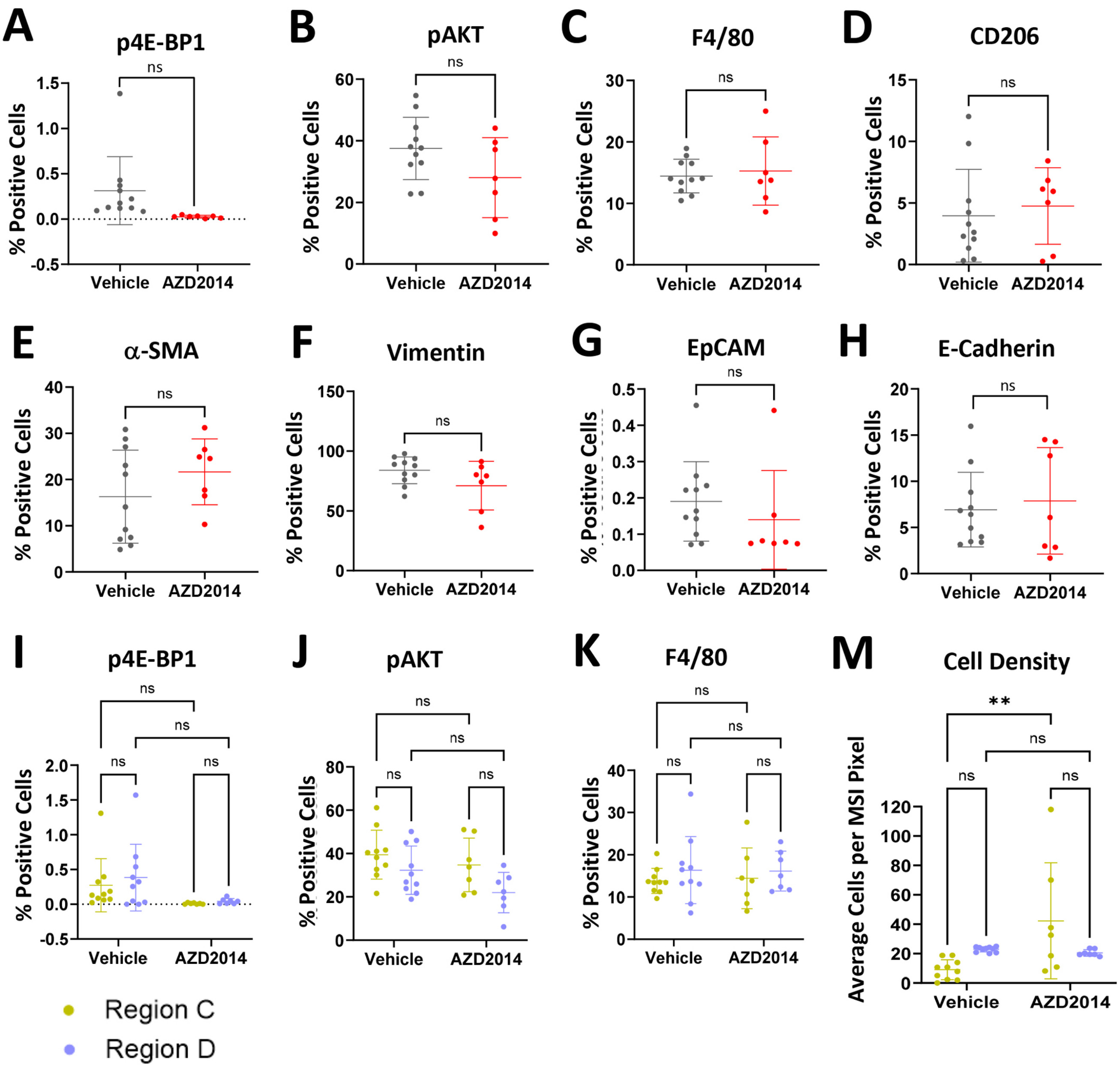

Fig. S6. Example endpoints from Imaging Mass Cytometry with no significant difference. For (A) p4EBP1 and (b) pAKT, (C) F4/80 positive macrophages (D) CD206 positive cells, (E) aSMA or (F) vimentin, (G) EpCam, (H) E-cadherin at a total tissue are assessment with comparing control and AZD2014 treatment. For (I) p4EBP1, (J) pAKT, (K) F4/80 staining between region C and D. (M) Differences in cell density of the regions. NS not significant, *p≤0.05, *p≤0.01, *p≤0.001 in two-way ANOVA.

1st in vivo experiment – PD biomarker WB data

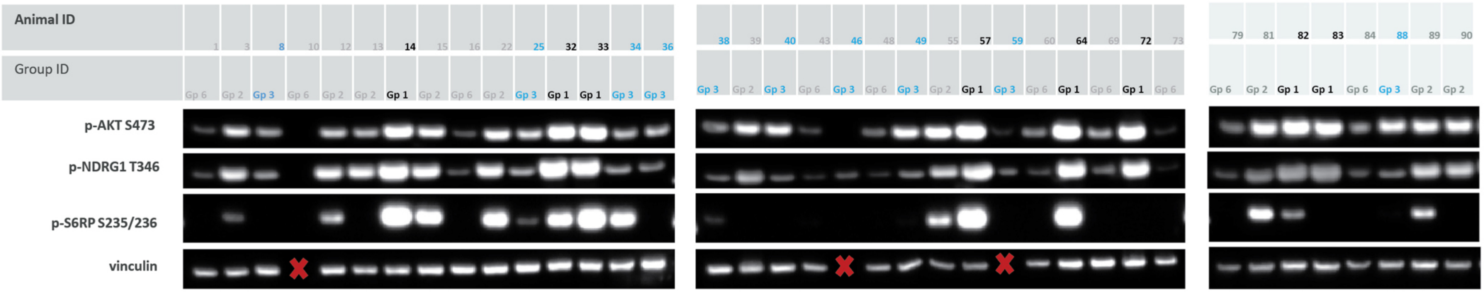

Relevant groups shown in graphical form in Fig. 1A are highlighted as below Blots were loaded in a random order Group 1 – vehicle control Group 3 – AZD2014

2nd in vivo experiment – PD biomarker WB data

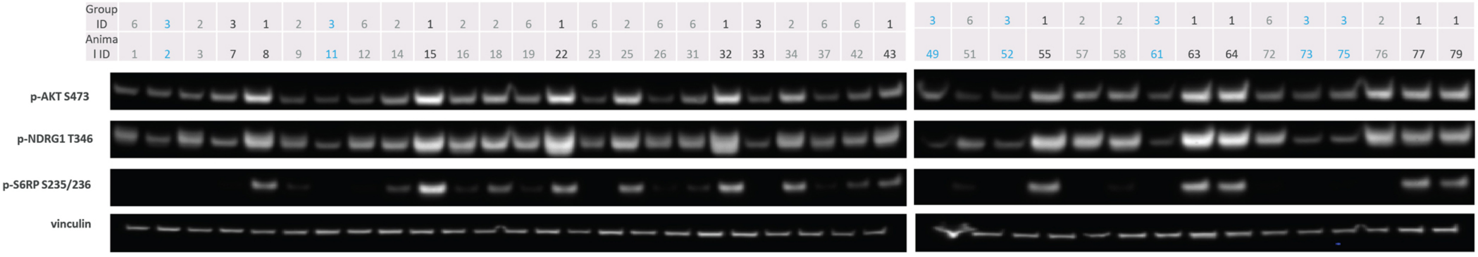

Relevant groups shown in graphical form in Figure 1A are highlighted as below Blots were loaded in a random order Group 1 – vehicle control Group 3 – AZD2014

3rd in vivo experiment – PD biomarker WB data

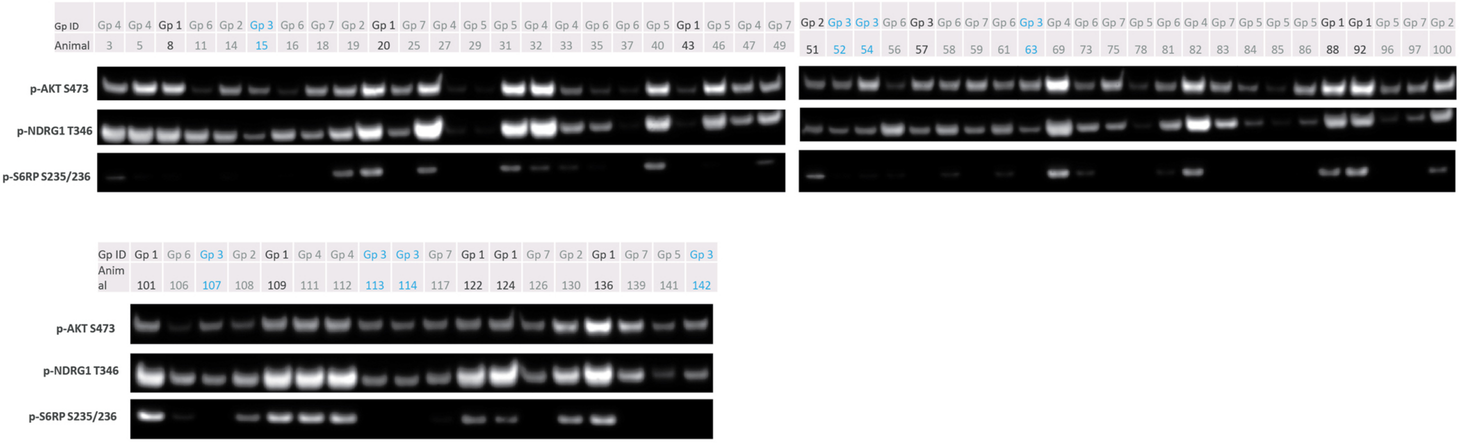

Fig. S7. Raw western blot images.

Disease Models & Mechanisms • Supplementary information

Table S1. Antibody panel used for Imaging Mass Cytometry analysis

Target	Clone	Metal tag	Product code
ATPase	EP1845Y	89Y	ab167390
αSMA	1A4	141Pr	3141017D
Cleaved Caspase 3	D3E9	142Nd	3142004A
Vimentin	D21H3	143Nd	3143027D
Collagen I	Polyclonal	144Nd	AB758
CD68	FA-11	145Nd	MCA1957GA
Cleaved Caspase 3	E83-77	147Sm	ab208003
Pan-CK	C11	148Nd	3148020D
p4E-BP1	236B4	149Sm	3149023D
CD103	AF1990	150Nd	AF1990
Ly6G	1A8	151Eu	3151010B
CD11c	D1V9Y	153Sm	97585BF
CD11b	M1/70	154Sm	3154006B
F4/80	CI:A3-1	155Gd	MCA497GA
CD163	TNKUPJ	156Gd	14-1631-82
E Cadherin	24E10	158Gd	3158029D
pNDRG1	D98G11	159Tb	5482BF
GLUT1	EPR3915	160Gd	ab196357
pAKT (Ser473)	D9E	162Dy	4060BF
FOXOa	75D8	163Dy	2497BF
pERK (Thr202/Tyr204)	20G11	164Dy	4376BF
CD31	390	165Ho	3165013B
EpCam (CD326)	G8.8	166Er	3166014B
Tenascin C	Polyclonal	167Er	AB19011
Ki67	B56	168Er	3168001B
CD206	CD68C2	169Tm	3169021B
HMGCS1	D1Q9D	170Yb	42201BF

Disease Models & Mechanisms • Supplementary information

Target	Clone	Metal tag	Product code
pERK1/2	D13.14.4E	171Yb	3171021D
pS6	N7-548	172Yb	3172008A
γH2AX	JBW301	173Yb	05-636
MHCII (I-A/I-E)	M5/114.15.2	174Yb	3174003B
Arg1	Polyclonal	175Lu	AF5868
pHH3	HTA28	176Yb	3176024D

Table S2. Raw data for metabolites detected

Available for download at
https://journals.biologists.com/dmm/article-lookup/doi/10.1242/dmm.050804#supplementary-data
